# Primary CNS lymphoproliferative disease, mycophenolate and calcineurin inhibitor usage

**DOI:** 10.18632/oncotarget.5292

**Published:** 2015-09-16

**Authors:** Genevieve M. Crane, Helen Powell, Rumen Kostadinov, Patrick Tim Rocafort, Dena E. Rifkin, Peter C. Burger, Richard F. Ambinder, Lode J. Swinnen, Michael J. Borowitz, Amy S. Duffield

**Affiliations:** ^1^ Department of Pathology, The Johns Hopkins Medical Institutions, Baltimore, MD, USA; ^2^ Department of Biostatistics, Johns Hopkins Bloomberg School of Public Health, Baltimore, MD, USA; ^3^ Division of Hematologic Malignancies, Sidney Kimmel Comprehensive Cancer Center (SKCCC) at Johns Hopkins, Baltimore, MD, USA; ^4^ Department of Pharmacy Practice and Science, University of Maryland School of Pharmacy, Baltimore, MD, USA; ^5^ Veterans’ Affairs Healthcare System and Division of Nephrology, Department of Medicine, University of California-San Diego, San Diego, CA, USA

**Keywords:** post-transplant lymphoproliferative disorder, primary central nervous system lymphoma, Epstein-Barr virus, mycophenolate mofetil, calcineurin inhibitors

## Abstract

Immunosuppression for solid organ transplantation increases lymphoproliferative disease risk. While central nervous system (CNS) involvement is more rare, we noticed an increase in primary CNS (PCNS) disease. To investigate a potential association with the immunosuppressive regimen we identified all post-transplant lymphoproliferative disease (PTLD) cases diagnosed over a 28-year period at our institution (174 total, 29 PCNS) and all similar cases recorded in a United Network for Organ Sharing-Organ Procurement and Transplant Network (UNOS-OPTN) data file. While no PCNS cases were diagnosed at our institution between 1986 and 1997, they comprised 37% of PTLD cases diagnosed from 2011–2014. PCNS disease was more often associated with renal vs. other organ transplant, Epstein-Barr virus, large B-cell morphology and mycophenolate mofetil (MMF) as compared to PTLD that did not involve the CNS. Calcineurin inhibitors were protective against PCNS disease when given alone or in combination with MMF. A multivariate analysis of a larger UNOS-OPTN dataset confirmed these findings, where both MMF and lack of calcineurin inhibitor usage were independently associated with risk for development of PCNS PTLD. These findings have significant implications for the transplant community, particularly given the introduction of new regimens lacking calcineurin inhibitors. Further investigation into these associations is warranted.

## INTRODUCTION

Post-transplant lymphoproliferative disease (PTLD) is a well-known complication of solid organ transplantation with both type and level of immunosuppression associated with risk for developing lymphoproliferative disease. Renal transplant recipients have been reported to have a relative risk of 12.6 of developing PTLD compared to the general population of developing lymphoma, while transplants requiring a higher level of immunosuppression, such as liver or combined heart/lung, have a relative risk from 30 to as high as 240 [1]. The type of immunosuppression has been shown to alter PTLD risk, most strikingly with treatment with anti-thymocyte globulin (ATG) or OKT3 for induction therapy or treatment of acute episodes of rejection. Patients who received ATG or OKT3 had an increased PTLD risk of approximately 9-fold [1–5] compared to those who did not, which may in part be secondary to increased levels of immunosuppression. However, drug mechanism may also play a role as anti-IL-2 receptor antibodies, when used in equivalent clinical situations as OKT3 and ATG, were as effective in reducing graft loss [3] but were not associated with increased PTLD risk [1, 3].

PTLD may involve any organ with central nervous system (CNS) involvement in 10–15% of cases [1, 6–8]. For example, a study of the Collaborative Transplant Study database from 1985–2001 found CNS involvement in 11.7% of PTLDs arising in kidney transplant recipients and only 3–4% of the PTLDs in liver, heart or combined heart/lung transplant recipients [1]. More commonly, PTLDs arose in the graft itself, the gastrointestinal tract or presented as disseminated disease [1, 8].

In our practice we noticed an apparent increase in the number of diagnoses of primary CNS (PCNS) PTLD. As immunosuppressive therapy has advanced, the association between the immunosuppressive drug regimen and the site at which lymphoproliferative disease develops has not been extensively investigated. We performed a retrospective review of all PTLDs diagnosed at our institution over the past 28 years and analyzed publicly available data collected by the United Network for Organ Sharing (UNOS) and Organ Procurement and Transplant Network (OPTN).

## RESULTS

### Patient demographics

We identified 177 cases of PTLD (Table 1); 88 were primary pathologic diagnoses made on surgical pathology specimens, 8 were primary pathologic diagnoses made at the time of autopsy and 78 were sent from other institutions for consultation with the pathologists at our institution. Six of the patients with a diagnosis of PTLD on a surgical pathology or consult specimen also subsequently underwent autopsy at our institution (Johns Hopkins Hospital; JHH). A total of 29 patients represented PCNS disease. An additional 3 patients with secondary involvement of the CNS from a systemic PTLD were identified, but due to their small number and the involvement of both sites, they were excluded from further analysis. The remaining 145 cases were non-CNS PTLDs.

**Table 1:** Demographics of PTLD patients from the JHH and UNOS-OPTN datasets

	Non CNS PTLD	PCNS PTLD
**JHH PTLD**		
Total cases	145	29
Male	88 (61%)	14 (48%)
Female	57	15
Median age (yrs)	47 (29–58)	60 (50–68)
Adult	121	29
Pediatric	24 (14%)	0 (0%)
Transplant to diagnosis (yrs)	3.5 (0.8–7.9)	3.8 (2.5–6.9)
Status (deceased)	68 (47%)	14 (48%)
Diagnosis to death (yrs)	1.1 (0.2–4.7)	1.2 (0.4–4.1)
**UNOS-OPTN Dataset**		
Total cases	6882	84
Male	4507 (66%)	50 (60%)
Female	2375	34
Median age	47 (25–57)	50 (36–62)
Adult	5485	77
Pediatric	1397 (20%)	7 (8%)
Transplant to diagnosis	4.1 (1.1–8.3)	3.8 (1.2–8.4)
Diagnosis to death	9.5 (0.9–15.9)	3.0 (0.4–11.7)
Deceased	3435 (50%)	50 (60%)
Alive	2904 (42%)	32 (38%)
Lost to follow-up	294 (4%)	2 (2%)
Re-transplant	249 (4%)	0 (0%)
Ethnicity		
White	5286 (77%)	58 (69%)
Black	659 (10%)	12 (14%)
Hispanic	647 (9%)	6 (7%)
Other	290 (4%)	8 (10%)
HLA mismatch	4 (3–5)	4 (3–5)
Rejection Episode*		
Yes	148 (6%)	1 (4%)
Yes but not treated	48 (2%)	0 (0%)
No	2160 (92%)	26 (96%)

All patients with a PTLD diagnosis at our institution (JHH) between 10/1986 and 8/2014 were identified and categorized as having PCNS or non-CNS disease. An additional 3 patients had PTLD involving both the CNS and extracranial sites and were excluded from this analysis. The demographics from patients with a PTLD diagnosis as reported in the UNOS-OPTN database from 10/1999 to 4/2014 are shown. For both datasets, values are reported as median and interquartile range (IQR) in years where applicable. If the patient received multiple solid organ transplants, time from transplant to diagnosis was measured from the most recent transplantation event. The HLA mismatch score was recorded on a scale of 0 to 6. UNOS-OPTN datasets from individual solid organ transplant types were analyzed and combined to eliminate duplicate data from patients who received multiple solid organ transplants. Percentages are calculated as relative to the total number of cases except for the percentage of rejection episodes, which was calculated as relative to the total number of patients for whom data was available in this category (indicated by ‘*’).

The most common indications for kidney transplant among patients who developed PTLD were diabetes(9), hypertension(7), glomerulnephritis(6), obstructive uropathy(5) and systemic lupus erythematosus(5). Except for glomerulonephritis(6), IgA nephropathy(3) and cystinosis(2), which were seen only in non-CNS PTLD patients, primary diseases requiring transplant were similar between PCNS and non-CNS PTLD patients (Table 2).

**Table 2:** Underlying primary disease diagnoses as indication for transplant in PTLD patients diagnosed at JHH

		PCNS	Non-CNS
**Heart**	Cardiomyopathy	0	9
	Congenital	0	1
	Viral myocarditis	0	2
	Adriamycin	0	1
	Unknown	1	5
**Liver**	Biliary atresia	0	9
	HCV	0	4
	HBV	0	2
	Congenital/metabolic	0	3
	PBC	0	4
	PSC	0	5
	Other	1	5
	Unknown	0	2
**Lung**	Cystic fibrosis	0	3
	IPF	0	3
	Congenital	0	1
	Scleroderma	0	1
	Other	0	1
	Unknown	0	1
**Kidney**	DM	3	6
	Hypertension	1	6
	Glomerulonephritis	0	6
	IgA nephropathy	0	3
	Obstructive uropathy	2	3
	SLE	3	2
	PCKD	2	2
	FSGS	1	2
	Cystinosis	0	2
	Other	1	6
	ESRD NOS	2	8
	Unknown	12	14
**Multiple**	DM	1	4
	Other	0	4

All multi-organ transplants in this series included a renal transplant. Abbreviations: hepatitis C (HCV), hepatitis B (HBV), hepatocellular carcinoma (HCC), primary biliary cirrhosis (PBC), primary sclerosing cholangitis (PSC), idiopathic pulmonary fibrosis (IPF), diabetes mellitus type I or II (DM), polycystic kidney disease (PCKD), focal segmental glomerulosclerosis (FSGS), end stage renal disease not otherwise specified (ESRD NOS).

**Figure 1:**
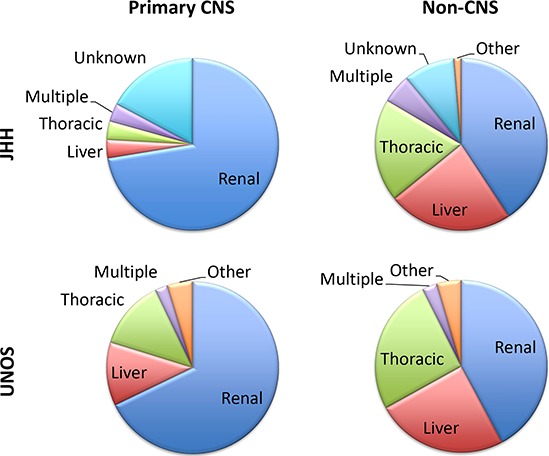
Distribution of PTLD cases according to type of transplanted organ for both JHH and UNOS-OPTN datasets PCNS PTLD was more strongly associated with renal transplant than expected based on the number of renal transplants performed (59% of all solid organ transplants) with a similar trend in both the JHH and UNOS-OPTN datasets. Non-CNS PTLD occurred more often in liver and thoracic transplant patients including heart, lung and combined heart/lung recipients, similar to previous reports [1].

PCNS and non-CNS patients were similar regarding median age at diagnosis, time from transplant to diagnosis and mortality (Table 1). Even though the majority of all PTLD cases were associated with renal transplant, renal transplant patients nonetheless had a particular predilection for PCNS PTLD. Based on nationwide UNOS-OPTN data, renal transplants comprise 59% of the total solid organ transplants performed; however, 75% of PCNS PTLD patients had a renal transplant compared to 47% of non-CNS PTLD patients (Figure 1).

### Incidence of PCNS PTLD

**Figure 2:**
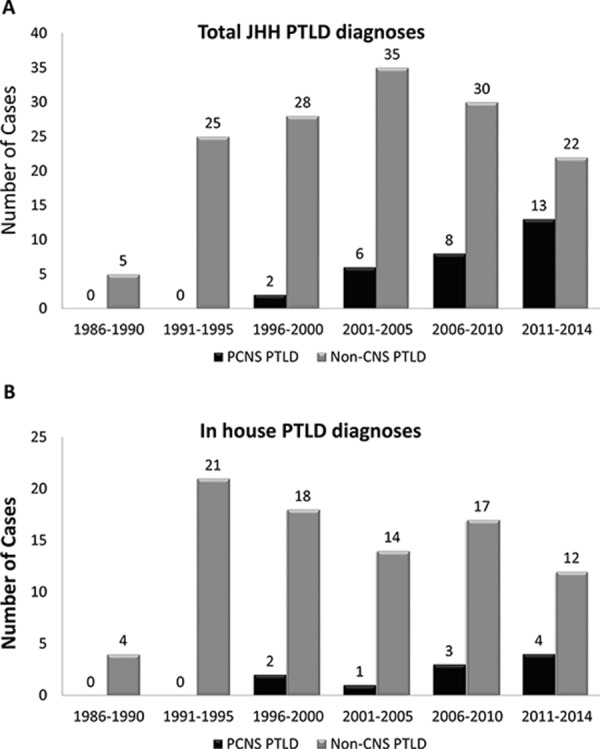

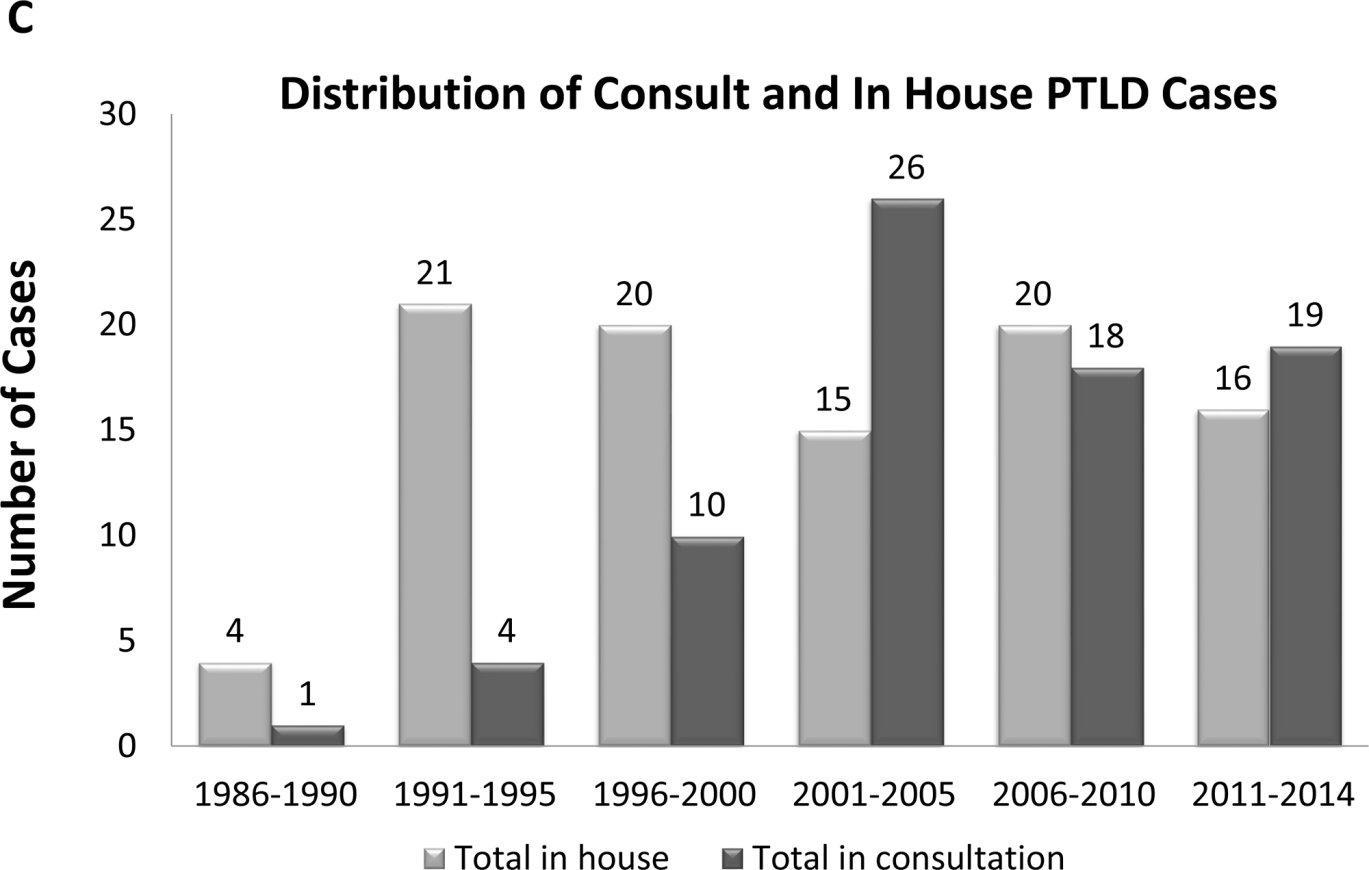
The incidence of PCNS lymphoproliferative disease is rising While the number of PTLD cases seen in consultation also increased since 1995, they have remained stable over the past 15 years. Evaluation of all PTLD cases diagnosed over a 28-year period at our institution (10/1986–8/2014) demonstrated a rise in the absolute and relative incidence of PCNS lymphoproliferative disease compared to non-CNS sites **A.** A similar trend was observed when considering only in house (surgical pathology and autopsy) PTLD cases **B.** While the number of PTLD cases seen in consultation also increased since 1995, they have remained stable over the past 15 years **C.** Two of the 3 patients with secondary involvement of the CNS from a systemic PTLD were diagnosed between 1986 and 1997 and one between 1998 and 2014. No rise in secondary CNS involvement of PTLD was identified in this study.

The number of PCNS PTLD cases diagnosed increased markedly over the period of study. While no PCNS cases were diagnosed at our institution between 1986 and 1997, 37% of PTLD cases diagnosed between 2011 and 2014 were PCNS PTLD (Figure 2). The proportion of PCNS PTLD cases diagnosed was 4.4-fold higher in the decade from 2005–2014 compared to the previous decade 1995–2004 with a two sample porportion test yielding a z-score of 3.9 (*p* < 0.0001). While many of these cases were seen in consultation, a similar trend was observed when only PTLD diagnoses made on in house surgical and autopsy specimens were considered (Figure 2), although it did not reach statistical significance (*p* = 0.16). Given the increase in the percentage of PCNS PTLDs in relation to all PTLD diagnoses, which have remained relatively constant over the last 15 years, these findings are not merely a function of an increase in the number of transplants performed. Similarly, the number of total PTLD cases received in consultation has not increased over the past 15 years (Figure 2). Combined with the trend toward increased PCNS PTLD diagnoses on in house specimens, it is unlikely these findings simply represent bias due to changing patterns of cases at a tertiary referral center.

### Pathologic classification

**Figure 3:**
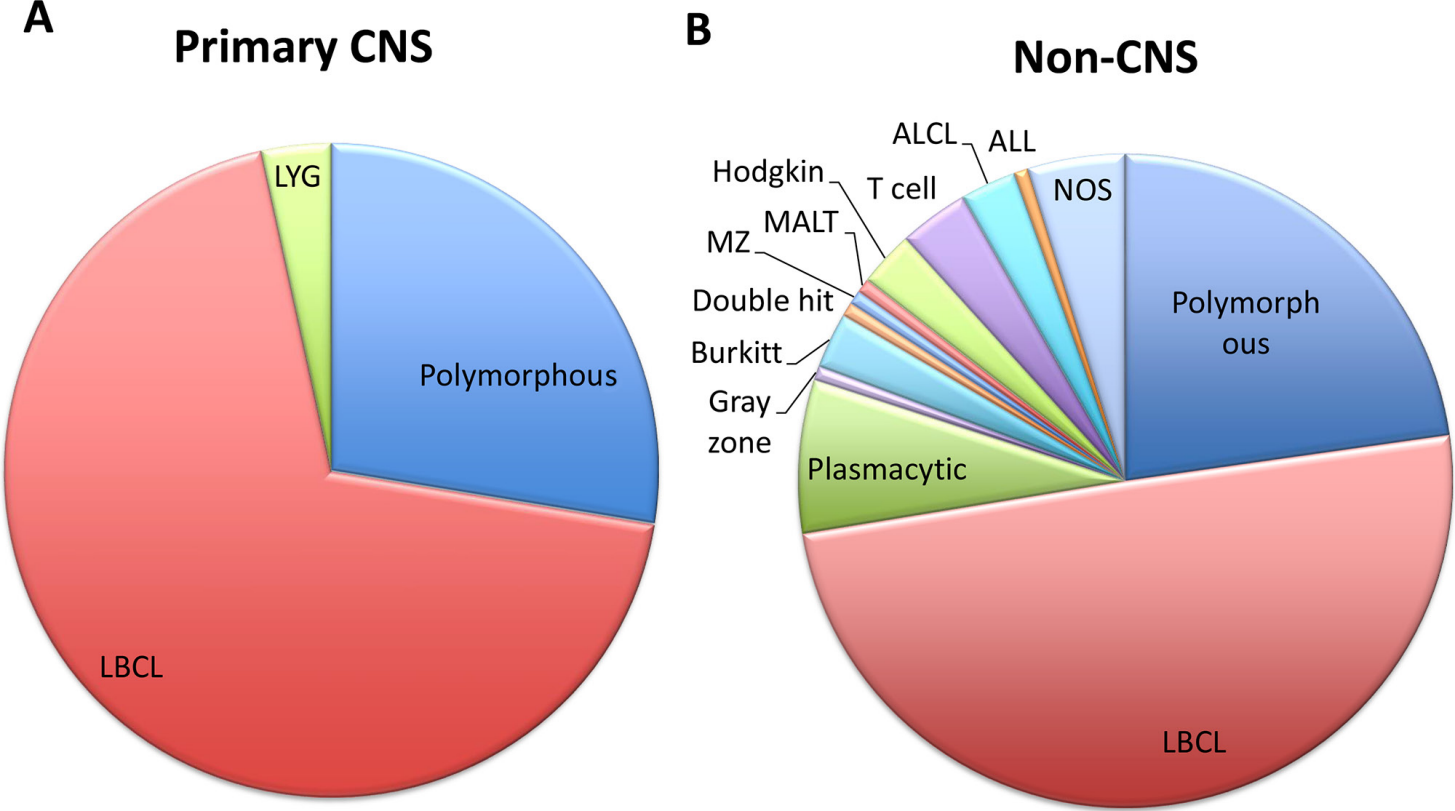
Primary CNS and non-CNS PTLD were predominantly large B-cell lymphomas Seventy-two percent of PCNS PTLD were monomorphic, including one case of lymphomatoid granulomatosis (LyG), an aggressive and angiodestructive form of B-cell lymphoma, and the remainder were large B-cell lymphomas (LBCL), **A.** Non-CNS PTLDs were 77% monomorphic, within which they were more morphologically diverse compared to PCNS PTLD **B.** While LyG was not seen in the set of non-CNS PTLD cases in this series, it has been reported in the post-transplant setting with either systemic or primary CNS involvement [49, 50]. Not otherwise specified (NOS) refers to diagnoses rendered as “PTLD” or “atypical lymphocytic proliferation consistent with PTLD” where the slides were not available for further subclassification. Additional abbreviations: anaplastic large cell lymphoma (ALCL) and acute lymphoblastic leukemia (ALL).

**Figure 4:**
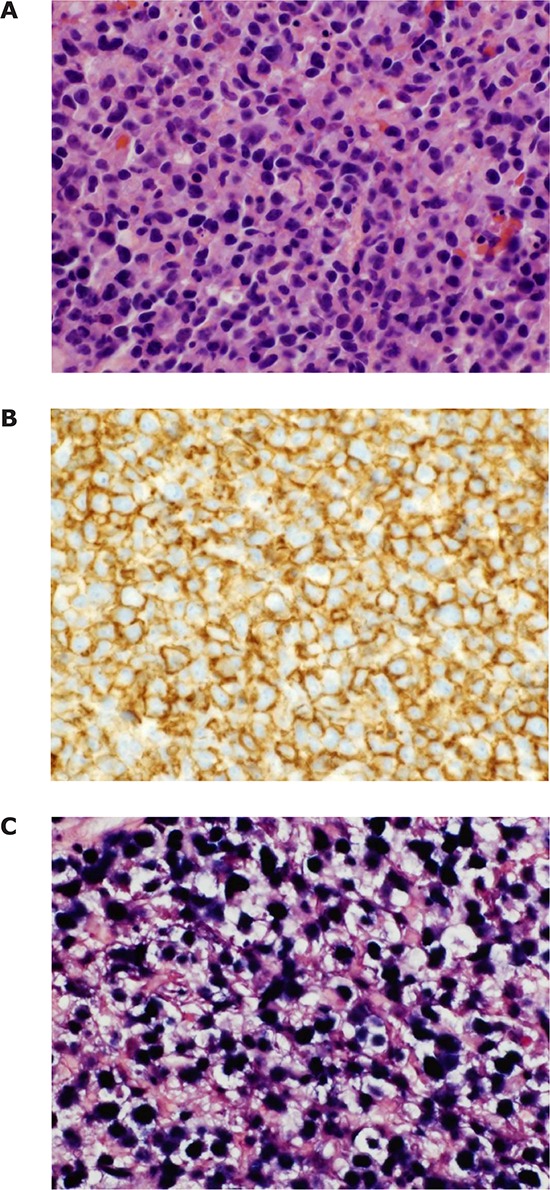
Histopathologic features of a monomorphic PCNS PTLD with large B-cell morphology This PTLD arose in a patient on a more recently introduced renal transplant regimen, including belatacept and MMF, designed to be given in the absence of CNIs. The brain parenchyma is diffusely replaced by a cellular infiltrate with atypical lymphoid cells that are predominantly large cells with vesicular chromatin and have a sheet-like growth pattern (**A.** H&E, 400X) with surrounding necrosis (not shown). The atypical cells are diffusely positive for CD20 (**B.** 400X) and EBV as detected by *in situ* hybridization for EBV-Barr virus encoded small RNAs (EBER). (**C.** 400X).

Both PCNS and non-CNS PTLD were predominantly classified as monomorphic PTLD (72% PCNS, 77% non-CNS), and most of the classifiable lymphomas were large B-cell lymphomas (Figure 3). Histopathologic features of a typical PCNS large B-cell neoplasm arising in a renal transplant recipient are shown (Figure 4). Lymphoproliferative disorders arising outside of the CNS were more morphologically diverse and included Burkitt lymphoma, anaplastic large cell lymphoma, angioimmunoblastic T-cell lymphoma and peripheral T-cell lymphoma amongst others. Two low-grade non-CNS lymphomas were also identified in post-transplant patients, both with a marginal zone lymphoma phenotype. Although not formally considered PTLD by WHO criteria [9], they were included in this analysis as extra-nodal MALT-type lymphomas have previously been reported in the post-transplant setting [10]. There were 4 cases of systemic lymphoma with secondary involvement of the CNS. Three of these cases were monomorphic, systemic PTLDs with large B-cell morphology. The fourth case was a diagnosis of human T-cell lymphotrophic virus 1 (HTLV-1)- associated adult T-cell leukemia/lymphoma in a renal transplant recipient that secondarily involved the CNS. Given the uncertain relationship of HTLV-1-associated lymphoproliferative disease with immunosuppression [11], this patient was not included as a diagnosis of PTLD.

**Figure 5:**
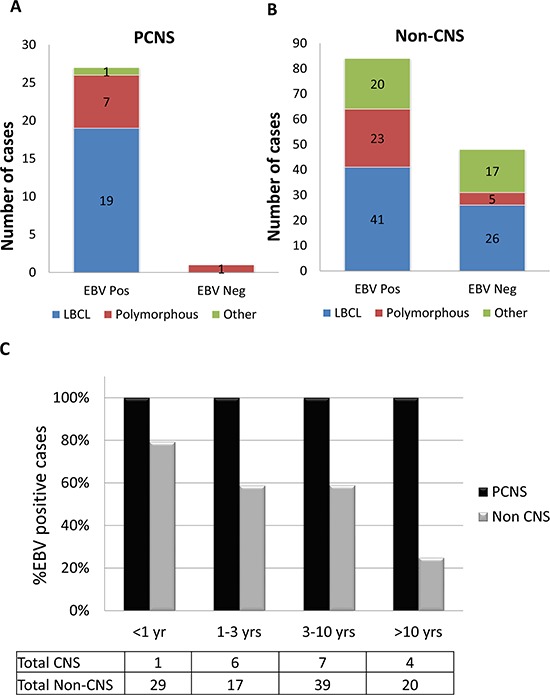
PCNS PTLD was more strongly associated with EBV than disease arising within other sites Nearly all PCNS PTLD cases were EBV-positive (96%), **A.** compared to 64% of cases arising in non-CNS sites **B.** (Chi square test, *p* < 0.005). Differences in EBV status between PCNS and non-CNS cases were not attributable to differences morphologic type. Where time from transplant was known, PCNS PTLDs were EBV-associated, while in non-CNS cases, the fraction of EBV-negative cases increased with time from transplant **C.** The total number of cases of each PTLD for which both time from transplant and EBV status were known is tabulated.

Irrespective of morphologic type, PCNS PTLD was associated with EBV (27/28) compared to non-CNS PTLD (84/132, Chi-squared test 10.2, *p* < 0.005, Figure 5). The fraction of EBV-negative PTLDs diagnosed increased with time from transplant in non-CNS cases. By contrast, all but one PCNS PTLD was EBV-associated. EBV data were available for only 1 of 3 cases of systemic PTLD that involved the CNS, which was an EBV-positive large B-cell lymphoma. One of the 2 non-CNS marginal zone lymphomas was EBV-positive, in contrast to the prior reported series of extranodal low-grade MALT-type lymphomas in the post-transplant setting in which all 5 cases were EBV-negative [10].

### Association with immunosuppressive regimen

**Figure 6:**
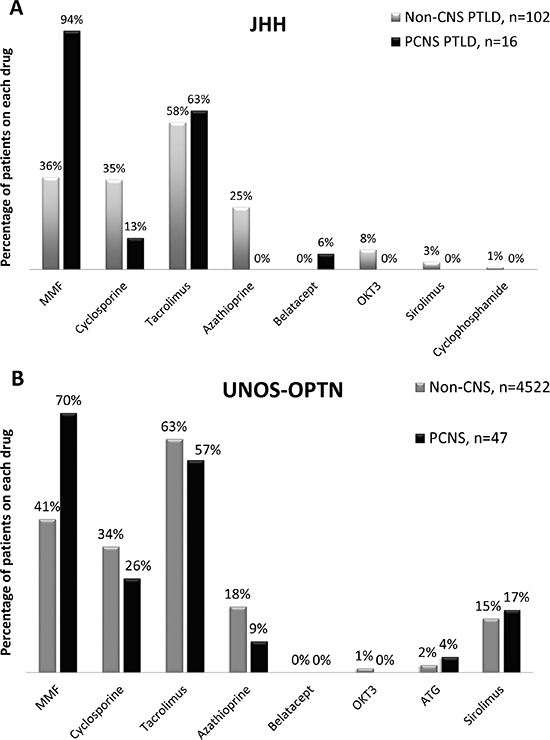
Drugs included in the immunosuppressive regimens of JHH and UNOS-OPTN patients JHH PTLD patients who developed PCNS PTLD **A.** were significantly more likely to be taking MMF (15/16) compared to patients who developed non-CNS PTLD (37/102, *p* < 0.0001). Analysis of a larger UNOS-OPTN datafile also identified an association with inclusion of MMF in the immunosuppressive drug regimen and PCNS compared to non-CNS disease **B.** Percentages are greater than 100% as the majority of patients were on multi-drug regimens. The total number of patients with drug data available is indicated. Data for less common drugs were not tabulated. The majority of patients were taking prednisone prior to diagnosis (not shown).

Compared to patients who developed non-CNS PTLD, PCNS patients were more likely to have been taking MMF (15/16) in the year prior to and/or at the time of diagnosis (37/102 non-CNS, OR 41, 95% CI 5.3 to 324, *p* < 0.001, Figure 6). None of the 3 patients with secondary CNS involvement of a systemic PTLD were taking MMF prior to or at the time of diagnosis. Notably, these 3 patients received their transplants in 1986, 1994 and 1995, and MMF was FDA approved for use in solid organ transplantation in 1995. Thus, there is no evidence of an increase in secondary CNS PTLD since widespread adoption of MMF for transplantation.

**Figure 7:**
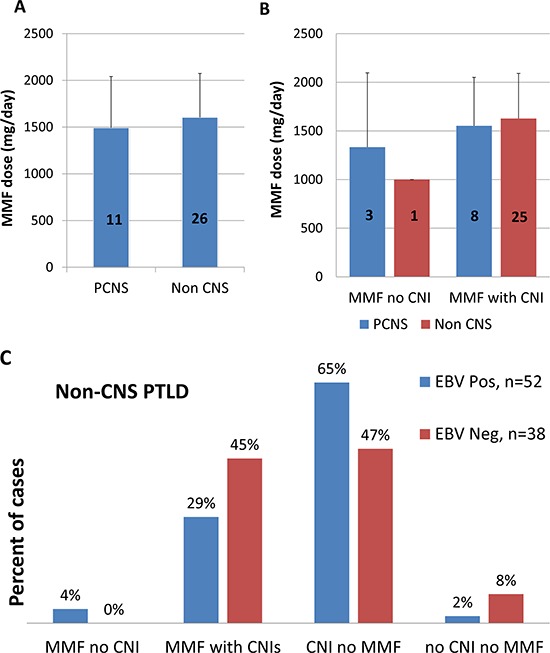
Standard MMF dosing did not vary between patient groups JHH patients who developed PCNS versus non-CNS PTLD did not differ significantly in the dose of MMF that they received **A.** Similarly, MMF dosing did not differ significantly between patients who received MMF without CNIs compared to MMF with CNIs in either PCNS or non-CNS sites **B.** The number of cases for which data were available is indicated on each bar. Both recommended and recorded MMF dosing in children differed substantially from that recommended for immunosuppression in the adult. Children were, therefore, excluded from this portion of the analysis. This resulted in the exclusion of 4 children (ages 2–10) from the non-CNS cases, and zero children from the PCNS cases. While both EBV and MMF were significantly associated with PCNS PTLD, no clear association between the drug regimen and EBV was identified in non-CNS PTLD patients **C.** There was no evidence for a protective effect of CNIs against the development of EBV-associated PTLD in non-CNS sites.

Patients whose immunosuppressive regimens contained CNIs had a significantly lower incidence of PCNS PTLD: PCNS disease comprised 66.7% of the PTLD cases that arose in patients on regimens with MMF but not CNIs, but only 1.7% of the PTLD diagnoses in patients on CNIs but not MMF (Table 3). This translated to an 118-fold higher odds for patients on MMF without CNIs to develop PCNS compared to non-CNS PTLD than patients on CNIs alone (95%CI, 8.7–1597; *p* < 0.001). Patients who received MMF and CNIs together had an intermediate risk of developing PCNS disease, with an 18-fold increased odds compared to patients on CNIs alone (95% CI, 2.3–150; *p* < 0.01), suggesting a partial protective effect of CNIs against the development of PCNS PTLD. There was no evidence to suggest a dose effect of MMF as a factor in these differences. Standard dosing regimens are recommended at our institution for adult patients, although they may be adjusted based on individual side effects or other factors. In adult patients where data were available, MMF dosing was similar between patients that developed PCNS and non-CNS PTLD and between patients who were taking MMF regimens with or without CNIs (Figure 7).

**Table 3:** MMF is associated with PCNS disease and CNIs are protective

			%PCNS
JHH PTLD	MMF/no CNI	*N* = 6	66.7%
	MMF/any CNI	*N* = 46	23.9%
	No MMF/any CNI	*N* = 60	1.7%
	No MMF/tacrolimus	*N* = 29	0%
	No MMF/cyclosporine	*N* = 31	3.1%
	No MMF/No CNI	*N* = 6	0%
UNOS-OPTN	MMF/no CNI	*N* = 70	11.4%
	MMF/any CNI	*N* = 1477	1.3%
	No MMF/any CNI	*N* = 1500	0.5%
	No MMF/tacrolimus	*N* = 1014	0.7%
	No MMF/cyclosporine	*N* = 463	0.2%
	No MMF/No CNI	*N* = 126	0%

**Figure 8:**
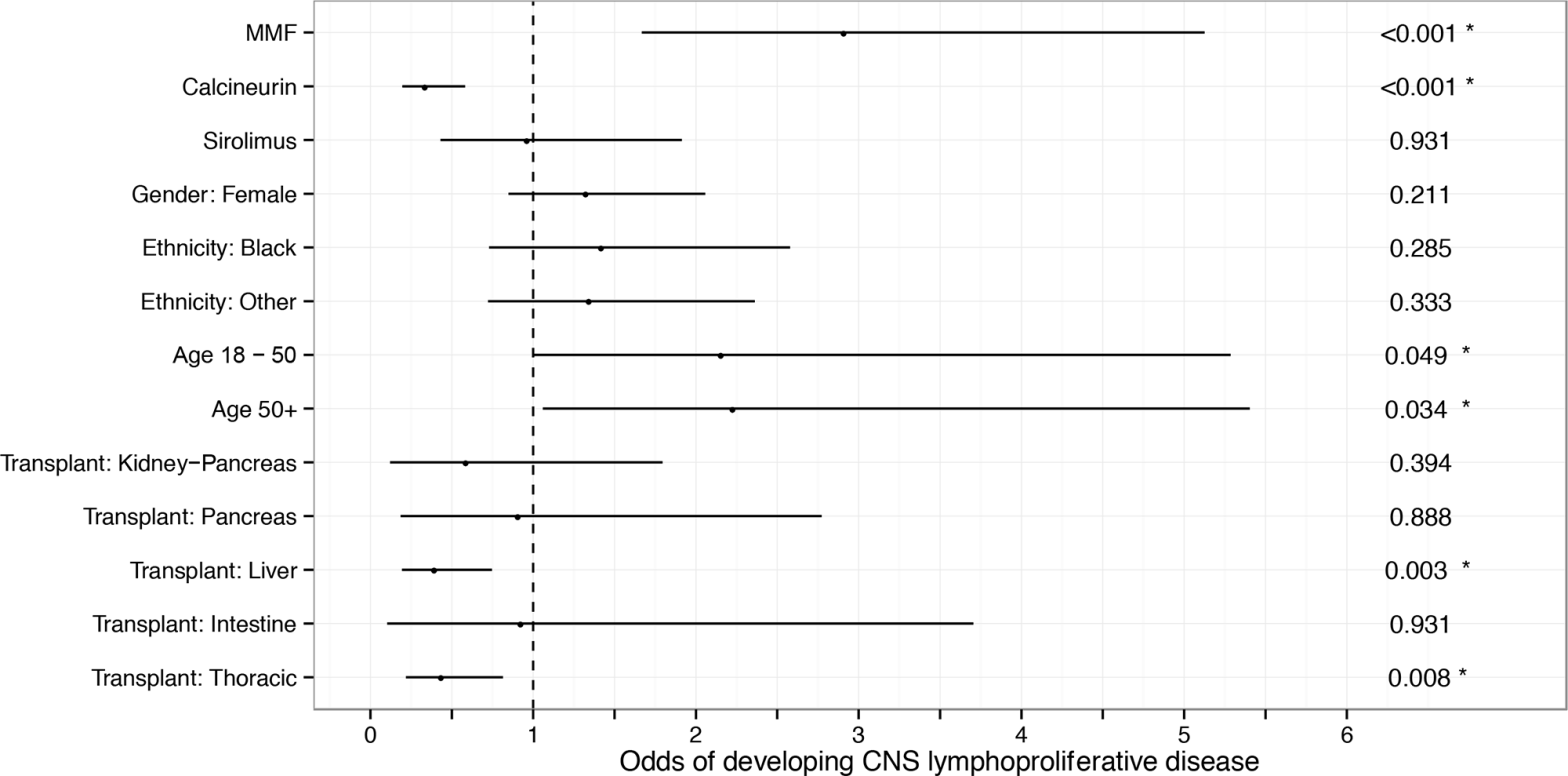
A logistic regression model of the UNOS-OPTN data demonstrates a protective effect of CNIs against PCNS PTLD and an independent association of PCNS PTLD with MMF Using Firth's penalized regression method, the odds of PCNS disease were significantly higher for patients taking MMF compared to those not taking MMF regardless of whether they were taking a CNI or their age, sex or transplant type. Patients taking CNIs had significantly reduced odds of PCNS PTLD compared to patients not taking CNIs. Evaluation of transplant type demonstrated significantly reduced odds of PCNS PTLD in liver and thoracic transplant recipients compared to kidney recipients. Age was divided into categories of age < 18, age 18–50 and age > 50 with age 18–50 or > 50 conferring borderline increased risk of PCNS PTLD compared to age < 18. Gender and ethnicity differences did not alter odds of PCNS PTLD.

To partially exclude effects of multidrug regimens, patients on only MMF, MMF and CNIs or only CNIs (+/– prednisone) were compared. The percentage of cases for each regimen that were PCNS disease is indicated for both the JHH and UNOS-OPTN datasets. When given in combination with MMF, CNIs had a protective effect against the development of PCNS PTLD. Nonetheless, the majority of both PCNS and non-CNS PTLDs still arose in patients on regimens containing CNIs as they are included in most standard drug regimens. A fraction of immunosuppressive regimens did not contain either MMF or CNIs (“No MMF/No CNI”), of which the most common medications were azathioprine, cyclophosphamide and sirolimus with or without steroids. A formal logistic regression analysis was also performed confirming these findings (Figure 8).

While nearly all PCNS PTLD were associated with both MMF treatment and EBV, no direct relationship was seen between MMF, CNIs and EBV in non-CNS PTLD (Figure 7). For example, in non-CNS PTLDs, 17/34 cases were EBV-associated in patients taking MMF and 35/56 cases were EBV-associated in patients not taking MMF (OR, 0.6; 95% CI 0.3–1.4; *p* = 0.25).

### Analysis of the UNOS-OPTN dataset

A second analysis was performed using a larger, UNOS-OPTN STAR data file that included a total of 6966 PTLD cases, of which 4569 had associated immunosuppression data. While UNOS-OPTN data collection does not routinely include PTLD site, searching additional text fields identified 84 patients who were specifically recorded as having primary CNS involvement. Both PCNS and non-CNS cases were more common in men than women (66% and 60%, respectively), which reflected the distribution seen in the overall transplant population (62%). The distribution of total solid organ transplant recipients according to ethnicity was 65% white, 18% black and 11% Hispanic with a similar distribution seen in both PCNS and non-CNS PTLD patients (Table 1). No differences between PCNS and non-CNS PTLD patients in the degree of human leukocyte antigen (HLA) mismatch or the frequency of recorded rejection episodes were identified. Changes in the incidence of PCNS PTLD with time were more difficult to evaluate in the UNOS-OPTN dataset than our pathology database as PTLD data fields were not recorded until 1999, and there was a lag between diagnosis and data reporting. Nonetheless, characteristics of the PCNS PTLD patients that were identified could be compared to the large set of total transplant recipients with a recorded PTLD diagnosis.

As in the JHH dataset, patients in the UNOS-OPTN dataset who developed PCNS PTLD were more likely to have been taking MMF than patients who developed non-CNS PTLD (Figure 6). PCNS PTLDs were over-represented in the fraction of PTLDs that arose in patients on MMF without CNIs (11.4%) compared to PTLDs that arose in patients on CNIs without MMF (0.53%, Table 3). Patients on MMF without CNIs had a 24-fold increased risk of developing PCNS than non-CNS PTLD than patients on CNIs without MMF (95% CI, 7.5–76; *p* < 0.001). In addition, patients on MMF without CNIs were more likely to develop PCNS than non-CNS disease as compared to patients on MMF with a CNI (OR, 9.9; 95% CI, 3.6–25; *p* < 0.001), demonstrating a protective effect of CNIs against PCNS PTLD. Nonetheless, the majority of both PCNS and non-CNS PTLDs still arose in patients on regimens containing CNIs as these are the most common transplant regimens. Both CNIs (tacrolimus and cyclosporine) had a similar protective effect. In the absence of MMF, the odds of developing PCNS compared to non-CNS PTLD were unaffected by whether cyclosporine or tacrolimus was taken (OR, 3.2; 95% CI, 0.4–145; *p* = 0.45).

Kidney transplant recipients represented the highest fraction of PTLD patients within the UNOS-OPTN dataset (42%); however, similar to the JHH dataset, renal transplant recipients were relatively over-represented in PCNS PTLD (70%, Figure 1).

Analysis of the UNOS-OPTN dataset using Firth's penalized regression method showed that the odds of PCNS PTLD were significantly increased when taking MMF as compared to not taking MMF (odds 2.9; 95% CI, 1.7–5.1; *p* < 0.002; Figure 8). CNIs significantly reduced the odds of PCNS disease compared to not taking CNIs (odds 0.34; 95% CI, 0.20–0.58; *p* < 0.001). In addition, independent of the immunosuppressive regimen used, the odds of developing PCNS PTLD was significantly lower for both liver (odds 0.40; 95% CI, 0.19–0.75; *p* = 0.003) and thoracic (odds 0.44; 95% CI, 0.22–0.81; *p* = 0.008) transplants compared to kidney transplant. Gender (female compared to male) and ethnicity (black or other compared to white) did not significantly alter odds of developing PCNS PTLD in this model, although there was a borderline increase in odds of PCNS PTLD among patients aged 18–50 or > 50 compared to those < 18.

## DISCUSSION

The question of a possible association between PCNS PTLD with MMF and other new biologic agents has emerged from small case series [12] as well as clinical trial data [13]. Additional evidence emerged in a larger French study, where Snanoudj et al. characterized PTLDs arising in patients who received kidney transplants between 1976 and 1998 and found that 6 of 10 patients diagnosed with PCNS PTLD > 3 years post-transplant had been recently switched from azathioprine to MMF [14]. A multi-center study of 84 PCNS PTLD patients by Evens et al. found that MMF was the most common immunosuppressant prior to diagnosis (69 of 84 patients), and 21 of them were on MMF as a monotherapy [15]. While these findings were intriguing, MMF use is common and no statistical comparison was made to a non-CNS PTLD group. Furthermore, to our knowledge a protective role of CNIs against developing PCNS lymphoproliferative disease has not previously been suggested in the literature.

We identify a rise in the incidence of PCNS PTLD since 1986, both in the absolute number of PCNS diagnoses and in the percentage they comprise of the total PTLD cases (Figure 2). While many of these cases were seen in consultation following the arrival of a surgical neuropathologist at our institution in 1995 (PCB), a similar trend is observed when only new diagnoses of inpatient specimens are considered. In addition, the number of consult cases with a diagnosis of PTLD has not increased over the past 15 years. Similarly, Snanoudj et al. found 18 of 25 PCNS PTLD cases identified in their series were diagnosed after 1997. This was postulated to relate to the level of immunosuppression or other factors [14]; however, our data demonstrate a strong association between PCNS disease and the immunosuppressive regimen. The risk of developing PCNS PTLD compared to other sites was higher for patients on MMF-containing regimens and was especially high in patients on MMF who were not also taking CNIs. Analysis of a larger UNOS-OPTN dataset confirmed these findings, and in a multivariable analysis of this dataset, both MMF and lack of CNI usage were independently associated with risk for development of PCNS PTLD.

In the 1960s and 70s following the widespread adoption of renal transplantation and development of successful immunosuppression regimens with azathioprine and prednisone, PCNS PTLD was the most common type of PTLD observed [16, 17]. For example, in one large study at that time, slightly more than half of the patients with PTLD had PCNS disease [18]. A similar increased risk of PCNS PTLD was documented in multiple other studies during this time period [19–22]. In addition, the immunosuppression associated with untreated HIV/AIDS has been associated with a several thousand-fold increased risk of PCNS lymphoma compared to the general population [23, 24]. While several studies that compared regimens containing CNIs or azathioprine to MMF found MMF was associated with a similar if not lower overall PTLD risk [1, 3, 17, 25, 26], they did not consider the relative occurrence of PCNS PTLD compared to PTLD arising in other sites.

Taken together, these findings suggest the CNS may have an inherent susceptibility to lymphoproliferative disease in the context of immunosuppression, and the specific immunosuppressive regimen affects the likelihood of PCNS disease. CNIs, when given alone or in combination with MMF, appear to protect against the development of PCNS PTLD. Thus, our observed rise in the incidence of PCNS PTLD is likely not only due to immunosuppression with MMF but also due to a decline in the usage of the protective CNIs. This interpretation would explain why, in both our dataset and the UNOS database, only a small fraction of the PTLDs that developed in patients on CNIs in the absence of MMF involved the CNS. In addition, the rarity of PCNS PTLD diagnoses in the late 1980s and 1990s both in the literature [2, 14] and in the JHH dataset appears to correspond to the introduction and widespread adoption of cyclosporine for transplantation in 1983. Indeed, one early study following the introduction of cyclosporine found that PTLDs arising following immunosuppression with cyclosporine were more likely to be widespread, involve lymph nodes or the gastrointestinal tract and rarely involve the brain compared to then standard azathioprine-based regimens [27].

Given the potential of CNIs for nephrotoxicity and challenges associated with dosing, regimens including MMF and newer biologic agents without CNIs have become increasingly popular in the renal transplant setting [13]. While this trend may help explain why the PCNS cases in this series were significantly more associated with renal transplants than non-CNS PTLDs, thoracic and liver transplant were still associated with a signficantly lower odds of PCNS PTLD compared to kidney in the logistic regression model. The association of PCNS PTLD with renal transplant has also been observed by other investigators [6, 7, 15]. Additional factors associated with renal transplant and propensity for developing PCNS PTLD may, therefore, warrant further investigation.

Level of immunosuppression is also a significant risk factor for the development of lymphoproliferative disease. PCNS lymphoma in HIV only develops with profound immunosuppression [28], and the overall risk of PTLD is increases with level of immunosuppression [1]. This raises questions regarding whether patients on MMF alone compared to MMF in combination with CNIs may have had different levels of immunosuppression related to differences in MMF dosing. However, where MMF dosing was available, no differences were identified between these groups (Figure 7). In addition, JHH comprehensive transplant center guidelines indicate the same MMF dose in adult patients, and all patients were treated within those guidelines.

That said, the immunosuppressive regimen may be scaled back due to signs of over-immunosuppression or symptoms concerning for PTLD. If CNIs were discontinued in such patients, the patients could appear to be taking only MMF at the time of diagnosis. In the JHH dataset, however, all 4 PCNS PTLD patients who were taking MMF in the absence of CNIs had been taking this as a maintenance immunosuppressive regimen for at least a year prior to diagnosis as verified in the clinical records. Maintenance drug regimens both at and prior to diagnosis were similarly included in the analysis of the UNOS-OPTN dataset. Strikingly, Evens et al. also found 21 of 84 patients with PCNS PTLD were taking MMF alone [29], although the frequency and timing of MMF use as a single agent in this population was not stated.

While drug dosing data was not available in the UNOS-OPTN dataset, acute rejection was used as a surrogate for depth of immunosuppression because occurrence of acute rejection is typically followed by a period of increased immunosuppression. We saw no differences in the incidence of acute rejection between patients who developed PCNS and non-CNS PTLD. Moreover, there was no difference in the HLA matching of the transplanted organ between PCNS and non-CNS PTLD groups, where a greater number of mismatches correlates with increased risk of acute rejection [30, 31]. Together these data do not suggest the increased incidence of PCNS PTLD with MMF is related to an increased level of immunosuppression.

Similar to previous studies of PCNS disease in HIV and transplant populations [6, 7, 9, 14, 15, 32, 33], the vast majority of PCNS cases in our dataset were EBV-positive. Non-CNS cases were more morphologically diverse, but differences in EBV status were independent of tumor morphology. In non-CNS cases the fraction of EBV-negative PTLDs increased with time from transplant similar to previous reports [9, 34, 35], but PCNS cases were EBV-positive regardless of time from transplant. Overall, these findings demonstrate a tight correlation between PCNS PTLD and EBV infection and raise the possibility of a link between EBV lytic cycle activation and the drug mechanism of action. Indeed, PCNS PTLD has been associated with EBV lytic viral expression [33], and CNIs have been shown to inhibit the initial step of the EBV lytic cycle activation *in vitro* [36, 37]. In addition, CNIs are highly lipophilic resulting in more stable concentrations in the brain than the plasma [38], potentially explaining why CNIs may be more protective in the brain against EBV-related PTLD than other sites. In addition to potential disruption of EBV-lytic cycle activation, inhibition of calcineurin signaling may disrupt downstream signaling of nuclear factor of activated T cells (NFAT), a family of transcription factors key in regulation of the immune system and also implicated in the pathogenesis of diffuse large B-cell lymphoma [39, 40]. Intriguingly, NFAT-signaling also regulates non-hematopoietic cells, and inhibition of NFAT expression with cyclosporine A decreased tumorigenicty in bladder cancer cell lines as well as a mouse model of bladder cancer [41].

Alternatively, Fink et al. have recently demonstrated a pattern of EBV microRNA expression unique to some PCNS PTLDs, suggesting possible mechanistic differences in how EBV drives tumorigenesis in the CNS compared to non-CNS sites [33]. Other microRNAs may also be important in the regulation of EBV-related diffuse large B-cell lymphomas compared to those that are EBV-negative [42]. Further study is needed to determine how these differences may interact with the mechanism of immunosuppression to alter susceptibility to tumorigenesis.

It is as yet unclear whether mechanisms underlying EBV-associated lymphomagenesis will be the same between different forms of immunosuppression and immune dysregulation (e.g. primary immunodeficiency, HIV, transplant and the elderly). However, data thus far suggests that gene expression with EBV-associated lymphomas is not unique in elderly patients compared to patients younger than 50 years and common mechanisms may be involved [43].

Regardless of potential mechanism, this is the first study to demonstrate a statistically significant association between the immunosuppressive drug regimen and PCNS PTLD. Our findings suggest that the decline in use of CNIs following the introduction of MMF in 1995 may have unmasked an inherent susceptibility of the CNS for immunosuppression-related lymphoproliferative disease.

Identifying risk factors associated with PCNS lymphoproliferative disease is important given the challenges of CNS-directed therapy [6, 44] as well as the association of CNS disease with a more dismal prognosis in some earlier series, though not ours [6, 8, 45, 46]. Of note, this discrepancy in survival may relate to inclusion of patients with involvement of both CNS and extracranial sites in the earlier studies, which has been associated with a worse prognosis [6, 46, 47]. Survival of PCNS PTLD patients in this study (52% JHH, 40% UNOS-OPTN) was similar to that reported for other more recent large, multi-center evaluations of PCNS PTLD (34–43%) [7, 15]. Regardless of these potential differences, both CNS and non-CNS disease are associated with significant mortality in this study as well as others [1, 6, 7, 15, 29, 48] and efforts to limit the incidence of all immunosuppression-related lymphomas are warranted.

Given the wide variability in immunosuppression regimens between institutions, these findings highlight the need to initiate multi-center trials to optimize graft function while minimizing toxicity and PTLD risk. Further investigation into potential drug interactions and strategies for the prevention of PCNS PTLD are warranted.

## MATERIALS AND METHODS

Following institutional review board approval, the Johns Hopkins Hospital pathology database was searched from 10/1986 to 8/2014 to identify all in-house, autopsy and consultation cases for patients with a diagnosis of PTLD who had received a solid organ transplant. Bone marrow transplant patients were excluded as the majority were for a primary hematolymphoid disorder. Clinical, morphologic and phenotypic characteristics of these neoplasms were recorded where available, including patient age, sex, type and reason for transplant, date of transplant in relation to the timing of diagnosis, immunosuppressive regimen and survival. In all cases, the maintenance immunosuppressive regimen for one year prior to the onset of patient PTLD symptoms was verified and included in the analysis. A similar search was performed on a UNOS Standard Transplant Analysis and Research (STAR) file using OPTN data on all solid organ transplant recipients from 10/25/99 (when PTLD-specific data fields were first included) to 4/14/14.

Odds ratios with 95% confidence intervals (CI) were calculated by the Fisher's exact test to compare the association of PCNS and non-CNS disease with the drug regimen. Chi-squared tests were used to test associations with other parameters. Evaluation of PCNS compared to non-CNS PTLD incidence over time was done using a one-tailed, two-sample proportion *z*-test. A logistic regression analysis on the UNOS-OPTN dataset was performed using Firth's penalized logistic regression. It was necessary to use a penalization due to the small number of identifiable cases of PCNS PTLD within the UNOS dataset. The regression included whether the drug regimen at the time of diagnosis or prior to diagnosis contained sirolimus, mycophenolate mofetil (MMF), or calcineurin inhibitors (CNIs) tacrolimus or cyclosporine. Age, gender, ethnicity and transplant type were included as potential confounders.

## References

[1] Opelz G, Döhler B (2004). Lymphomas after solid organ transplantation: a collaborative transplant study report. American journal of transplantation : official journal of the American Society of Transplantation and the American Society of Transplant Surgeons.

[2] Opelz G, Henderson R (1993). Incidence of non-Hodgkin lymphoma in kidney and heart transplant recipients. Lancet.

[3] Cherikh WS, Kauffman HM, McBride MA, Maghirang J, Swinnen LJ, Hanto DW (2003). Association of the type of induction immunosuppression with posttransplant lymphoproliferative disorder, graft survival, and patient survival after primary kidney transplantation. Transplantation.

[4] Swinnen LJ, Costanzo-Nordin MR, Fisher SG, O'Sullivan EJ, Johnson MR, Heroux AL, Dizikes GJ, Pifarre R, Fisher RI (1990). Increased incidence of lymphoproliferative disorder after immunosuppression with the monoclonal antibody OKT3 in cardiac-transplant recipients. The New England journal of medicine.

[5] Caillard S, Dharnidharka V, Agodoa L, Bohen E, Abbott K (2005). Posttransplant lymphoproliferative disorders after renal transplantation in the United States in era of modern immunosuppression. Transplantation.

[6] Buell JF, Gross TG, Hanaway MJ, Trofe J, Roy-Chaudhury P, First MR, Woodle ES (2005). Posttransplant lymphoproliferative disorder: significance of central nervous system involvement. Transplantation proceedings.

[7] Cavaliere R, Petroni G, Lopes MB, Schiff D (2010). Primary central nervous system post-transplantation lymphoproliferative disorder: an International Primary Central Nervous System Lymphoma Collaborative Group Report. Cancer.

[8] Caillard S, Lelong C, Pessione F, Moulin B (2006). Post-Transplant Lymphoproliferative Disorders Occurring After Renal Transplantation in Adults: Report of 230 Cases From the French Registry. American Journal of Transplantation.

[9] Swerdlow SH, Campo E, Harris NL, WHO Classification of Tumours of Haematopoietic and Lymphoid Tissues1 (2008). WHO Classification of Tumours of Haematopoietic and Lymphoid Tissues.

[10] Hsi ED, Singleton TP, Swinnen L, Dunphy CH, Alkan S (2000). Mucosa-associated lymphoid tissue-type lymphomas occurring in post-transplantation patients. The American journal of surgical pathology.

[11] Nakamura N, Tamaru S, Ohshima K, Tanaka M, Arakaki Y, Miyauchi T (2005). Prognosis of HTLV-I-Positive Renal Transplant Recipients. Transplantation Proceedings.

[12] Sola-Valls N, Rodríguez C NY, Arcal C, Duran C, Oppenheimer F, Ribalta T, Lopez-Guillermo A, Campistol JM, Graus F, Diekmann F (2014). Primary brain lymphomas after kidney transplantation: an under-recognized problem?. Journal of nephrology.

[13] Grinyó J, Charpentier B, Pestana JM, Vanrenterghem Y, Vincenti F, Reyes-Acevedo R, Apanovitch AM, Gujrathi S, Agarwal M, Thomas D, Larsen CP (2010). An integrated safety profile analysis of belatacept in kidney transplant recipients. Transplantation.

[14] Snanoudj R, Durrbach A, Leblond V, Caillard S, Hurault De Ligny B, Noel C, Rondeau E, Moulin B, Mamzer-Bruneel M-F, Lacroix C, Charpentier B (2003). Primary brain lymphomas after kidney transplantation: presentation and outcome. Transplantation.

[15] Evens AM, Choquet S, Kroll-Desrosiers AR, Jagadeesh D, Smith SM, Morschhauser F, Leblond V, Roy R, Barton B, Gordon LI, Gandhi MK, Dierickx D, Schiff D, Habermann TM, Trappe R (2013). Primary CNS posttransplant lymphoproliferative disease (PTLD): an international report of 84 cases in the modern era. American journal of transplantation : official journal of the American Society of Transplantation and the American Society of Transplant Surgeons.

[16] Porter ka, marchioro tl, starzl te (1965). Pathological changes in 37 human renal homotransplants treated with immunosuppressive drugs. British journal of urology.

[17] Kauffman HM, Cherikh WS, McBride MA, Cheng Y, Hanto DW (2006). Post-transplant de novo malignancies in renal transplant recipients: the past and present. Transplant international : official journal of the European Society for Organ Transplantation.

[18] Hoover R, Fraumeni JF (1973). Risk of cancer in renal-transplant recipients. Lancet.

[19] Schneck SA, Penn I (1971). De-novo brain tumours in renal-transplant recipients. Lancet.

[20] Cho ES, Connolly E, Porro RS (1974). Primary reticulum cell sarcoma of the brain in a renal transplantation recipient. Journal of neurosurgery.

[21] Doak PB, Montgomerie JZ, North JD, Smith F (1968). Reticulum cell sarcoma after renal homotransplantation and azathioprine and prednisone therapy. British medical journal.

[22] Barnett LB, Schwartz E (1974). Cerebral reticulum cell sarcoma after multiple renal transplants. Journal of neurology, neurosurgery, and psychiatry.

[23] Besson C, Goubar A, Gabarre J, Rozenbaum W, Pialoux G, Châtelet FP, Katlama C, Charlotte F, Dupont B, Brousse N, Huerre M, Mikol J, Camparo P, Mokhtari K, Tulliez M, Salmon-Céron D (2001). Changes in AIDS-related lymphoma since the era of highly active antiretroviral therapy. Blood.

[24] Coté TR, Manns A, Hardy CR, Yellin FJ, Hartge P (1996). Epidemiology of brain lymphoma among people with or without acquired immunodeficiency syndrome. AIDS/Cancer Study Group. Journal of the National Cancer Institute.

[25] Robson R, Cecka JM, Opelz G, Budde M, Sacks S (2005). Prospective registry-based observational cohort study of the long-term risk of malignancies in renal transplant patients treated with mycophenolate mofetil. American journal of transplantation : official journal of the American Society of Transplantation and the American Society of Transplant Surgeons.

[26] Funch DP, Ko HH, Travasso J, Brady J, Kew CE, Nalesnik MA, Walker AM (2005). Posttransplant lymphoproliferative disorder among renal transplant patients in relation to the use of mycophenolate mofetil. Transplantation.

[27] Penn I, First MR (1986). Development and incidence of cancer following cyclosporine therapy. Transplantation proceedings.

[28] Kirk O, Pedersen C, Cozzi-Lepri A, Antunes F, Miller V, Gatell JM, Katlama C, Lazzarin A, Skinhøj P, Barton SE (2001). Non-Hodgkin lymphoma in HIV-infected patients in the era of highly active antiretroviral therapy. Blood.

[29] Evens AM, David KA, Helenowski I, Nelson B, Kaufman D, Kircher SM, Gimelfarb A, Hattersley E, Mauro LA, Jovanovic B, Chadburn A, Stiff P, Winter JN, Mehta J, Van Besien K, Gregory S (2010). Multicenter analysis of 80 solid organ transplantation recipients with post-transplantation lymphoproliferative disease: outcomes and prognostic factors in the modern era. Journal of clinical oncology : official journal of the American Society of Clinical Oncology.

[30] Schulman LL, Weinberg AD, McGregor C, Galantowicz ME, Suciu-Foca NM, Itescu S (1998). Mismatches at the HLA-DR and HLA-B loci are risk factors for acute rejection after lung transplantation. American journal of respiratory and critical care medicine.

[31] Trpkov K, Campbell P, Pazderka F, Cockfield S, Solez K, Halloran PF (1996). Pathologic features of acute renal allograft rejection associated with donor-specific antibody, Analysis using the Banff grading schema. Transplantation.

[32] Camilleri-Broet S, Davi F, Feuillard J, Seilhean D, Michiels JF, Brousset P, Epardeau B, Navratil E, Mokhtari K, Bourgeois C, Marelle L, Raphaël M (1997). AIDS-related primary brain lymphomas: Histopathologic and immunohistochemical study of 51 cases. Human Pathology.

[33] Fink SEK, Gandhi MK, Nourse JP, Keane C, Jones K, Crooks P, Jöhrens K, Korfel A, Schmidt H, Neumann S, Tiede A, Jäger U, Dührsen U, Neuhaus R, Dreyling M, Borchert K (2014). A Comprehensive Analysis of the Cellular and EBV-Specific MicroRNAome in Primary CNS PTLD Identifies Different Patterns Among EBV-Associated Tumors. American journal of transplantation : official journal of the American Society of Transplantation and the American Society of Transplant Surgeons.

[34] Leblond V, Davi F, Charlotte F, Dorent R, Bitker MO, Sutton L, Gandjbakhch I, Binet JL, Raphael M (1998). Posttransplant lymphoproliferative disorders not associated with Epstein-Barr virus: a distinct entity?. Journal of clinical oncology : official journal of the American Society of Clinical Oncology.

[35] Nelson BP, Nalesnik MA, Bahler DW, Locker J, Fung JJ, Swerdlow SH (2000). Epstein-Barr virus-negative post-transplant lymphoproliferative disorders: a distinct entity?. The American journal of surgical pathology.

[36] Liu S, Liu P, Borras A, Chatila T, Speck SH (1997). Cyclosporin A-sensitive induction of the Epstein-Barr virus lytic switch is mediated via a novel pathway involving a MEF2 family member. The EMBO journal.

[37] Goldfeld AE, Liu P, Liu S, Flemington EK, Strominger JL, Speck SH (1995). Cyclosporin A and FK506 block induction of the Epstein-Barr virus lytic cycle by anti-immunoglobulin. Virology.

[38] Butcher SP, Henshall DC, Teramura Y, Iwasaki K, Sharkey J (1997). Neuroprotective actions of FK506 in experimental stroke: *in vivo* evidence against an antiexcitotoxic mechanism. The Journal of neuroscience : the official journal of the Society for Neuroscience.

[39] Pham LV, Tamayo AT, Li C, Bueso-Ramos C, Ford RJ (2010). An epigenetic chromatin remodeling role for NFATc1 in transcriptional regulation of growth and survival genes in diffuse large B-cell lymphomas. Blood.

[40] Bruno A, Boisselier B, Labreche K, Marie Y, Polivka M, Jouvet A, Adam C, Figarella-Branger D, Miquel C, Eimer S, Houillier C, Soussain C, Mokhtari K, Daveau R, Hoang-Xuan K (2014). Mutational analysis of primary central nervous system lymphoma. Oncotarget.

[41] Kawahara T, Kashiwagi E, Ide H, Li Y, Zheng Y, Miyamoto Y, Netto GJ, Ishiguro H, Miyamoto H (2015). Cyclosporine A and tacrolimus inhibit bladder cancer growth through down-regulation of NFATc1. Oncotarget.

[42] Andrade TA, Evangelista AF, Campos AH, Poles WA, Borges NM, Camillo CM, Soares FA, Vassallo J, Paes RP, Zerbini MC, Scapulatempo C, Alves AC, Young KH, Colleoni GW (2014). A microRNA signature profile in EBV+ diffuse large B-cell lymphoma of the elderly. Oncotarget.

[43] Ok CY, Ye Q, Li L, Manyam GC, Deng L, Goswami RR, Wang X, Montes-Moreno S, Visco C, Tzankov A, Dybkaer K, Zhang L, Abramson J, Sohani AR, Chiu A, Orazi A (2015). Age cutoff for Epstein-Barr virus-positive diffuse large B-cell lymphoma - is it necessary?. Oncotarget.

[44] Heslop HE (2009). How I treat EBV lymphoproliferation. Blood.

[45] Phan TG, O'Neill BP, Kurtin PJ (2000). Posttransplant primary CNS lymphoma. Neuro-Oncology.

[46] Penn I, Porat G (1995). Central nervous system lymphomas in organ allograft recipients. Transplantation.

[47] Maecker B, Jack T, Zimmermann M, Abdul-Khaliq H, Burdelski M, Fuchs A, Hoyer P, Koepf S, Kraemer U, Laube GF, Muller-Wiefel DE, Netz H, Pohl M, Toenshoff B, Wagner H-J, Wallot M (2007). CNS or Bone Marrow Involvement As Risk Factors for Poor Survival in Post-Transplantation Lymphoproliferative Disorders in Children After Solid Organ Transplantation. Journal of Clinical Oncology.

[48] Nalesnik MA (2002). Clinicopathologic characteristics of post-transplant lymphoproliferative disorders. Recent Results Cancer Res.

[49] Seckin D, Barete S, Euvrard S, Frances C, Kanitakis J, Geusau A, Del Marmol V, Harwood CA, Proby CM, Ali I, Gulec AT, Durukan E, Lebbe C, Alaibac M, Laffitte E, Cooper S (2013). Primary cutaneous posttransplant lymphoproliferative disorders in solid organ transplant recipients: a multicenter European case series. Am J Transplant.

[50] Stravodimou A, Cairoli A, Rausch T, Du Pasquier R, Michel P (2012). PTLD Burkitt Lymphoma in a Patient with Remote Lymphomatoid Granulomatosis. Case Rep Med.

